# Fabrication and Applications of Microfluidic Devices: A Review

**DOI:** 10.3390/ijms22042011

**Published:** 2021-02-18

**Authors:** Adelina-Gabriela Niculescu, Cristina Chircov, Alexandra Cătălina Bîrcă, Alexandru Mihai Grumezescu

**Affiliations:** 1 Faculty of Engineering in Foreign Languages, University Politehnica of Bucharest, 011061 Bucharest, Romania; niculescu.adelina19@gmail.com; 2 Faculty of Applied Chemistry and Materials Science, University Politehnica of Bucharest, 011061 Bucharest, Romania; cristina.chircov@yahoo.com (C.C.); ada_birca@yahoo.com (A.C.B.); 3 Research Institute of the University of Bucharest—ICUB, University of Bucharest, 050657 Bucharest, Romania

**Keywords:** microfluidic devices, fabrication techniques, chip materials, biomedical applications, drug delivery, organ-on-a-chip

## Abstract

Microfluidics is a relatively newly emerged field based on the combined principles of physics, chemistry, biology, fluid dynamics, microelectronics, and material science. Various materials can be processed into miniaturized chips containing channels and chambers in the microscale range. A diverse repertoire of methods can be chosen to manufacture such platforms of desired size, shape, and geometry. Whether they are used alone or in combination with other devices, microfluidic chips can be employed in nanoparticle preparation, drug encapsulation, delivery, and targeting, cell analysis, diagnosis, and cell culture. This paper presents microfluidic technology in terms of the available platform materials and fabrication techniques, also focusing on the biomedical applications of these remarkable devices.

## 1. Introduction

According to George Whitesides, one of the most important personalities in this field, microfluidics represents “the science and technology of systems that process or manipulate small (10^–9^ to 10^–18^ L) amounts of fluids, using channels with dimensions of tens to hundreds of micrometers” [1,2,3]. Microfluidics evolved from the convergence of technologies and principles from several pre-existing domains, such as chemistry, physics, biology, material science, fluid dynamics, and microelectronics [1,4]. While the field of microfluidics, as such, is quite new, the concept of microchannels was previously integrated in the component capillaries of gas chromatography and capillary electrophoresis equipment (made of glass or quartz) or in flow reactors (made of metal) [2]. Consequently, more sophisticated structures for liquid flow guiding through microchannels started to be documented in patents from the 1980s [5]. Nevertheless, the debut of microfluidic technologies is considered to have been taken place in the 1990s, since when it has experienced exponential growth and become a powerful tool with enormous development potential [6,7,8,9].

Such miniaturized microscale devices are useful instruments for carrying out operations like reactions, separations, or the detection of various compounds [4,10]. Depending on their application and functional particularities, microfluidic devices can also be found in the literature as microreactors [3,6,11], lab-on-a-chip [12,13,14], or organ-on-a-chip [4,15,16]. Providing their destination use, microfluidic chips can be manufactured from a broad range of materials, employing diverse fabrication methods [17,18]. As many manufacturing techniques have already been presented in the literature and adopted in practice [10,19,20], the potential for advances in the field of microfluidics increases abruptly, bringing new perspectives to both the academic and industrial sectors [18]. Besides, this technology is promising for day-to-day applications, as several commercially available devices are already employed in pregnancy at-home-testing; virus (e.g., human immunodeficiency virus (HIV); Coronavirus Disease 19 (COVID-19); Herpes Simplex; and Hepatitis A, B, and C) rapid testing; and blood glucose monitoring [21,22].

The present review aims to thoroughly describe microfluidic technologies from the perspectives of chip fabrication in terms of material and method types and the main applications of the obtained devices.

## 2. Fabrication of Microfluidic Devices

### 2.1. Microfluidic Device Materials

One of the fundamental steps in microfluidic applications is selecting the optimum material for device fabrication [11]. Since, on a microscale surface, the properties are much more amplified, the platform material is likely to affect the properties of synthesized nanomaterials [2,6]. Specifically, unique phenomena emerge in capillary microfluidics due to shorter retention times, laminar flows, enhanced heat and mass transfer, and large surface-to-volume ratios [2]. Unlike for macroscale vessels, the wetting and contact angles of an aqueous solution on the chip materials are of fundamental importance [5]. Other essential properties that must be considered when choosing the material are durability, ease of fabrication, transparency, biocompatibility, chemical compatibility with the implied reagents, meeting the temperature and pressure conditions needed for the reaction, and the potential of the surface functionalization [11,23,24].

Various kinds of materials attempt to match these properties and can be used for the manufacturing of microfluidic devices [10,25]. Typical substrates include glass, silicon, metals, polymers, and ceramics, but the diversity and quality of materials are continuously increasing [6,11,17,24,25]. Each material has both advantages and disadvantages, depending on its destination use [6].

Metals present a series of advantages that make them suitable for microchip fabrication. They are cheap, widely accessible, easy to a machine; and can withstand high heat loads, high pressure, and toxic chemicals (except strong acids). Besides, their resistance to robust handling is convenient for cleaning operations [25,26,27]. The most popular metals for microfluidic devices are aluminum, copper, and iron, but they are most commonly found in alloys with other metals in order to fine-tune their chemical resistances [27]. Microfluidic devices made of metals have been demonstrated useful in nanomaterial synthesis, as size-tunable methacrylic nanoparticles were obtained in a stainless steel multi-lamination micromixer [26].

Silicon is among the first choices when it comes to microfluidic systems fabrication due to its ready availability, chemical compatibility, and thermostability [25,28]. The ease of fabrication, design flexibility, semiconducting properties, and the possibility of surface modifications provided enough reasons for silicon to be the dominant material for microfluidic platforms for decades [17]. However, several disadvantages must be considered when including this material in practical applications. The most evident limitation is the opacity of silicon, which renders it incompatible for optical detection in the visible and ultraviolet regions [2,10,25,28]. If in situ imaging is required, at least a portion of the device must be non-silicon [29]. Moreover, being quite fragile and having a high elastic modulus, incorporating active components, i.e., valves and pumps, in the silicon platform is complicated [17,28]. The price is also not in favor of silicon, as it is a relatively expensive material [10,25,28]. Nonetheless, silicon microfluidic platforms find use in biological applications, such as point-of-care medical diagnostics and organ-on-chip devices for drug toxicity screening [17].

Glass is chemically inert [10,17,28,30], thermostable [10,25,28], electrically insulating [2], rigid [28], biologically compatible [10,17], and allows easy surface functionalization [24,25,28]. These properties make glass-based microreactors suitable for carrying out chemical reactions that require extreme conditions: high temperatures, high pressures, and aggressive solvents [6,17,24,25]. The higher resolution at the micrometer scale that is achieved in glass microcapillary reactors compared with other materials makes these devices suitable for the better-controlled synthesis of emulsions and polymeric nanoparticles [26]. Compared to silicon, glass also has the advantages of excellent optical transparency [2,10,17], a lower price [28], and the possibility of integrating active components [17]. Not only valves and pumps of other materials (e.g., silicon, polymers, and hydrogel) can be employed in such chips, but the glass itself can be integrated as an active component in the form of ultra-thin glass sheets (0.004-mm in thickness) [31]. Due to its transparency, glass can be used for optical detection [25]. Additionally, the glass’s thermal and chemical stability allows for the effective cleaning of the device once the experiment is over, either by heating the chip or washing it with chemicals [30]. Additionally, glass is the traditional material for chemists and biologists, being chosen for most laboratory ware productions [25]. Glass compatibility with biological samples makes it useful for biochemical analyses [17]. Hence, it is no surprise that glass is one of the most popular materials for microfluidic chips [24]. In terms of composition, these substrates are usually made of soda-lime glass, borosilicate glass, and fused quartz [10]. However, microfabrication difficulties arise, limiting the applications of glass microfluidic devices. Despite being cheap, glass is expensive to be manufactured into chips, requires time-consuming labor, and preparation in cleanrooms is sometimes implied [10,28,29].

Low-temperature cofired ceramic (an aluminum oxide-based material) can be employed to fabricate microfluidic platforms [17,32]. The properties that recommend ceramics for microchips are their unique surface chemistry, good resistance to corrosive environments, and good stability at high temperatures [17,25]. Nevertheless, ceramics present some limitations in dimensional stability, porosity, and brittleness, making it difficult to integrate this type of material into a complete microsystem [25].

The versatility of polymers has attracted more and more attention to microfluidic device manufacturing, shifting away from silicon and glass chips. In comparison to inorganic materials, polymers are rather inexpensive and benefit from easier and lower-cost manufacturing techniques [2,10,25,26]. Polymer microfluidic platforms can be employed in a variety of applications, from nanoparticle synthesis to fluid manipulation [33]. Microreactors made of polymers are suitable for applications at either room temperature or higher temperatures (up to 200 °C), being quite useful for large-scale productions [6]. Additionally, transparent or semitransparent polymeric materials allow optical access to follow how the reaction advances, which is very important when nanocrystallizations are carried out [26]. The most common polymers utilized for microfluidic device fabrication include polydimethylsiloxane (PDMS), polymethylmethacrylate (PMMA), fluoropolymers, cyclo-olefin polymers and copolymers (COPs/COCs), and Thiol-ene polymers (TEs).

One of the most representative materials of this class is PDMS, an elastomer with excellent microchip fabrication properties [2,11]. PDMS is cheap [17,25], easy to mold [17,25], and good for prototyping [17], presenting optical transparency [11,17], gas permeability [2,11], biocompatibility [2,17], low autofluorescence [34], natural hydrophobicity [11], and high elasticity [2]. Owing to these properties, PDMS is valuable for bio-related research, such as long-term cell-culture, cell screening, and biochemical assays [2,34]. PDMS microdevices can detect bacteria, as well as their proteins and DNA, which is highly useful for disease diagnosis. Thin membranes made of PDMS can also be used for valves and pumps [17]. However, the same properties become obstacles in organic synthesis. The porosity of PDMS makes it an adsorptive material, in which many molecules can diffuse. This renders the material incompatible with organic solvents (e.g., hexane, toluene, and chloroform), as their molecules can be adsorbed into channel walls and swell the platform [2,9,17]. Another issue may arise from water evaporation through channel walls, which leads to a change in the concentration of a solution [2]. To overcome these problems, other polymeric materials are being investigated for microfluidic fabrication, depending on the desired properties and final application [11].

Another widely used material that is suitable for the manufacturing of microchips is PMMA [29,35]. PMMA is an amorphous thermoplastic [25,35] with slightly better solvent compatibility than PDMS [2] and no small-molecule absorption [29]. PMMA is optically transparent [17], has good mechanical properties [29], and allows surface modification [2] and prototyping at a small scale of production [17,35]. These properties are useful for a research setting, especially for organ-on-a-chip devices and micro-physiological systems [17,29].

Perfluorinated polymers, e.g., perfluoroalkoxy alkane (Teflon PFA), fluorinated ethylene-propylene (Teflon FEP), and polytetrafluoroethylene (PTFE), can be used for microfluidic devices due to their thermo-processability, chemical inertness, compatibility with organic solvents, and excellent antifouling properties [2,9,11]. Teflon presents optical transparency and a moderate permeability to gases and retains enough flexibility to make diaphragm valves. These materials are proposed for applications in cell cultures, high-precision assays, super-clean tools, and valve and pump fabrications [2,9,36]. PTFE is employed in synthesis devices, as it can tolerate a wide range of chemicals and temperatures up to 240 °C, being also naturally resistant to fouling channel blockage when aqueous solutions are involved due to its hydrophobic nature [11,26]. However, the examples of available fluoropolymer microdevices are sparse due to the lack of easy micropatterning and satisfactory elasticity [9].

COPs/COCs have recently attracted considerable research interest in microfluidics due to their favorable properties, such as optical transparency in the visible and near-UV spectrum, enhanced chemical resistance, low water absorptivity (<0.01%), good electrical insulating properties, long-term stability of surface treatments, and an extremely low level of impurities [25,29,37]. These thermoplastics are useful for synthesis devices in which aggressive solvents are employed, being able to withstand acids (e.g., hydrogen chloride, sulfuric acid, and nitric acid); bases (e.g., sodium hydroxide and ammonia); and polar solvents (e.g., ethanol and acetone) [37].

TEs are a family of macromolecular compounds consisting of two monomers, each with at least two thiols or allyl (or ene) groups. These materials could represent a better alternative to other polymers, as they have a significantly higher solvent resistance than PDMS, PMMA, and COCs. TEs also exhibit enhanced optical transparency in the visible spectrum, but the UV transmittance is composition-dependent [23].

Epoxy resins are another material used for microfluidic device fabrications. Although they are mostly used as a component in glass or silicon chips, these resins could also be used as the sole material in organ-on-a-chip devices, allowing the biological observation of cell growth [29,36]. As a thermosetting material, resins are endowed with enhanced stability at high temperatures, chemical resistance, transparency, and very high resolution with small features. However, the most striking disadvantage of these materials is their high cost [13,36].

Hydrogels are highly porous tridimensional networks of hydrophilic polymer chains that allow the diffusion of small molecules and bioparticles [2,17]. Other advantageous properties of hydrogels include their biocompatibility [2], low cytotoxicity [36], biodegradability [36], controllable pore size [2], high permeability [2], and aqueous nature [2]. Moreover, hydrogels resemble the extracellular matrix (ECM) [2], having intrinsic critical features to mimic natural mechanical and structural cues for cell adhesion, proliferation, and differentiation [38]. These characteristics make hydrogels ideal for encapsulating cells for 3D culture in tissue engineering research; for the delivery of solutions, cells, and other substances; and for sensors and actuators [2,36]. However, hydrogels are less frequently used as the primary fabrication material, because maintaining the device integrity is quite challenging and can limit their use in the long term [29]. Nonetheless, these materials can be employed in building microfluidic component functionalities, such as semipermeable barriers and smart valves, within a chip made of a more rigid material [17].

Since 2007, paper-based microfluidics has been explored as an alternative to expensive materials for microfluidic applications [34,39]. Paper-based systems benefit from simplicity [40], accessibility [29], significant low costs [29,39,40,41], high porosity [2,29], high physical absorption [24], ease of manipulation and sterilization [29], potential for chemical or biological modifications [29], similarity to the native ECM [29], bio-affinity [24], biocompatibility [29], light weights [41], and the ability to operate without supporting equipment [39,41]. What makes paper so special is the surface tension of a fluid and its contact angle with cellulose fibers [33]. Hence, the paper’s fluid flow is influenced mostly by the cohesive and adhesive forces that produce capillary action in the cellulose matrix [41,42,43]. Some researchers treat the pressure force in the analogy with electric circuits, comparing it to the electric voltage source that pushes fluid through the network [33]. Moreover, this phenomenon allows the precise guiding of fluids by hydrophobically modifying certain regions in the matrix [2]. The special behavior of paper and its advantageous properties make this material suitable for a variety of applications [34]. Paper-based systems are attractive for rapid point-of-care diagnostic testing and medical screening in the developing world [39,41,42], being mostly used together with a colorimetric or an electrochemical readout for detecting target biomolecules [24,42]. Besides, paper microfluidic systems can be directly and in situ operated, even in the absence of technical infrastructure and trained experts, making these devices a promising solution for field analysis or testing at home [40]. However, paper-based systems are limited by their poor mechanical strength in a wet state and thickness requirements for achieving transparency [29]. In addition, passive pumping may cause certain challenges concerning the precise design of the fluid circuit’s hydrodynamic resistance [43].

The materials previously mentioned can also be combined into hybrid devices to exploit their advantages in a synergic manner. In this respect, approaches that can be taken include placing soft films between hard chips in sandwich-like structures to form diaphragm valves, incorporating channels with substrates patterned with metal electrodes, combining several materials to adjust the channel permeability in specific regions, or implanting photocurable materials to obtain structures manufactured in situ [2]. One such composite microfluidic device was proposed by Koijc et al. [44]. They created a cost-effective chip that combined polyvinyl chloride (PVC) foils and Ceram Tapes. Their proposed device presented good optical, mechanical, and thermal characteristics and excellent resistance to high flow rates. Moreover, it benefited from the fact that each layer could be created and tested separately before lamination. Hence, high reliability and reproducibility could be achieved. Another strategy was approached by Gao et al. [45], who combined gas-permeable PDMS with Norland Optical Adhesive 81 (NOA81), a photocurable gas-impermeable polymer, to enable the local control of oxygen tension in microfluidic cell cultures. This hybrid device allowed researchers to establish hypoxic zones of precise dimensions and geometry inside microfluidic cell culture chambers. Chen et al. [46] took yet another approach, investigating microfluidic devices based on a glass–PDMS–glass sandwich configuration. The main advantage of such devices is the possibility to dismount and reuse them in various applications. Their proposed sandwich configuration could exceedingly increase the sealing strength of reversibly adhered devices, being also able to withstand high pressures.

By taking into account the above-discussed platform materials, Table 1 was comprised for better visualization of their features.

Recently, researchers have started to explore microdevices’ production by biodegradable polymers that can be orally ingested for drug delivery, imaging, and sensing. Materials such as polysaccharides (e.g., alginates, dextran, and chitosan) and protein-based polymers (e.g., gelatin) have been considered for these devices, yet they can be hardly scalable due to difficult processing and the high costs of extraction and purification from natural resources [49]. Nonetheless, several prototypes were designed. Abid et al. [50] fabricated asymmetric poly-ε-caprolactone (PCL) microcontainers loaded with paracetamol and coated with Eudragit^®^ S100. Their device protected the encapsulated drug until reaching the desired location in the small intestine. Another example was given by Zhou et al. [51], who designed a “microrocket” consisting of a poly (aspartic acid) (PASP) microtube, a thin Fe intermediate layer, and a core of Zn. The device was completely decomposed by the gastric acid or proteases found in the digestive tract.

### 2.2. Chip Fabrication Methods

**Figure 1:**
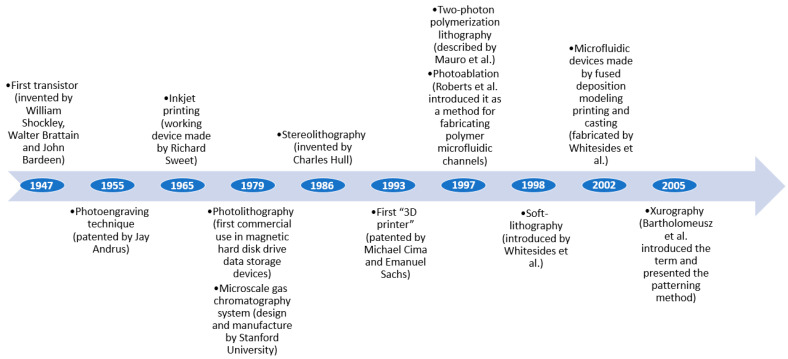
Important benchmarks in the evolution of microfluidic device fabrication. Created based on information from the literature references [52,53,54,55,56,57].

Microfluidics has come a long way in a relatively short time due to several technological advancements from various fields that have gained research interest and been adapted for chip production. Some of the most important breakthroughs that contributed to microfluidics development are comprised in Figure 1.

As previously described, there is a wide range of materials from which a microfluidic device can be fabricated. Each of these materials has different properties—hence, a different behavior when being processed. Therefore, fabrication methods must be adapted for the specific characteristics of the involved material and the finite product requirements. Another important aspect when choosing the fabrication technique is the cost. This is essential in microfluidic platforms because they are difficult-to-clean devices and are most often used as disposables. The chosen method must be economically feasible for one-time-only chips [58]. Moreover, for widespread adoption, chips should be manufactured in an accessible and scalable manner [59].

Fortunately, nowadays, many fabrication techniques have been described and adopted [54]. Waldbaur et al. offered a classification of these methods depending on how the microfluidic structure is created: by removing material (removing techniques) or by depositing material (depositing techniques) [19]. Another classification divides the fabrication methods depending on the nature of the processes involved—namely, chemical, mechanical, laser-based, and other processes [10]. Examples from each category are presented in Table 2.

#### 2.2.1. Chemical Processes

Several chemical fabrication processes have been used for a long time for manufacturing glass and silicon microfluidic channels [10,19]. The most commonly used chemical techniques are wet and dry etching and electrochemical discharge machining.

Wet etching has become popular due to the fast etching rate and the possibility of simultaneously processing a large quantity of wafers [10]. This technique requires strong chemicals for material removal, and the etchant of choice is usually hydrofluoric acid [62,63]. This represents a limitation of this fabrication technique, as highly corrosive etchants possess significant safety and environmental hazards [64]. Another disadvantage is the isotropic profile of the etched channels [10,62]. By contrast, dry etching techniques, also known as reactive ion etching, overcome some of the wet etching fabrication challenges. Such methods allow creating anisotropic, precise microscale channel profiles due to the directional nature of the ion bombardment [10]. Dry etching is recommended for transparent substrates but is otherwise not preferred because of its much slower rates compared to wet etching and poor selectivity relative to the mask [62].

Electrochemical discharge machining is a rather non-conventional fabrication process that uses an electrochemically generated spark on a tool surface. The spark is created by applying a voltage between two electrodes (one counter electrode and one tool electrode) immersed in an electrolyte. The high temperature of the spark removes the undesired material either thermally or chemically. The process can be applied to nonconductive materials, such as ceramics and glass [10].

#### 2.2.2. Mechanical Processes

Micromachining was one of the first known methods for fabricating microfluidic devices, as it was borrowed from the pre-existing field of semiconductors [20]. Mechanical processes must allow the production of crack-free surfaces while preserving good dimensional accuracy and surface roughness [10]. Such techniques are suitable for processing silicon and glass [65], but they can also be used for polymer-based devices to generate the replication master [20]. Methods such as mechanical cutting, abrasive jet machining, and ultrasonic machining benefit from low costs, high degrees of flexibility, and the possibility to be used together with other processes for creating complex 3D structures [10]. On the other hand, the main limitation of mechanical fabrication processes is their reduced precision and productivity compared to lithographic methods [10].

Micro-milling employs a high-precision computer numerical controlled motion system for removing bulk materials [18,66]. Unneeded materials are cut through intermittent contact with the workpiece by means of a rotary tool with two or more cutting edges. The method is simple, effective, precise, and economical, being suitable for creating complex 3D structures [10].

Additionally found in the literature as “blasting”, abrasive air-jet machining involves the injection of abrasive particles through a nozzle to be mixed with the air at high speed and very high pressure. Material removal is achieved by the kinetic energy of the particles colliding with the workpiece surface [10]. Similar to abrasive air-jet machining, abrasive water-jet machining is a nontraditional fabrication method that allows the production of complex 2D parts with tight accuracy [67]. The high viscosity of water, compared to air, provides better jet characteristics than in abrasive air-jet machining [10].

Ultrasonic machining also involves abrasives that model the workpiece through vibration. The working principle is based on creating microcracks on brittle materials, e.g., glass, silicon, and ceramics, leading to holes on the surface [10].

Xurography represents the patterning of an adhesive film through the use of a razor blade [57]. It was adapted as a low-cost technique for microfluidic chip fabrication, because it allows the production of robust, inexpensive devices in a short time (several minutes) and without requiring a cleanroom facility [57,66,68].

Injection molding is a common method for processing polymers for various objects of daily use [20]. Due to its high-throughput, cost efficiency, and precision, this method has also attracted interest in microfluidics fabrication [54,58]. Often found in the literature as micro-injection molding, this process consists of transferring pre-polymerized pellets of a thermoplastic from a hopper into a heated barrel. After the material melts, it is injected under pressure inside a heated mold cavity. The pressure is maintained for a given time, while the temperature is decreased below the polymer glass transition temperature. The solidified material is released from the mold [20,69]. Despite its advantages in terms of costs and method simplicity, the main limitations of micro-injection molding are material restrictions (only thermoplastics) and mold issues (expensive fabrication and limited resolution) [54].

Similar to injection molding, hot embossing is based on melting thermoplastics and shaping them into molds by means of pressure and heat [54]. However, instead of injecting the polymer into a cavity, the material is poured and pressed against the mold in such a way that the desired features are transferred from the cast to the softened polymer [18]. This difference allows stress reduction in the processed material. Moreover, more delicate designs can be obtained due to less shrinkage of the cast. However, the limitation of using only thermoplastics is also valid for this method [54]. 

**Figure 2:**
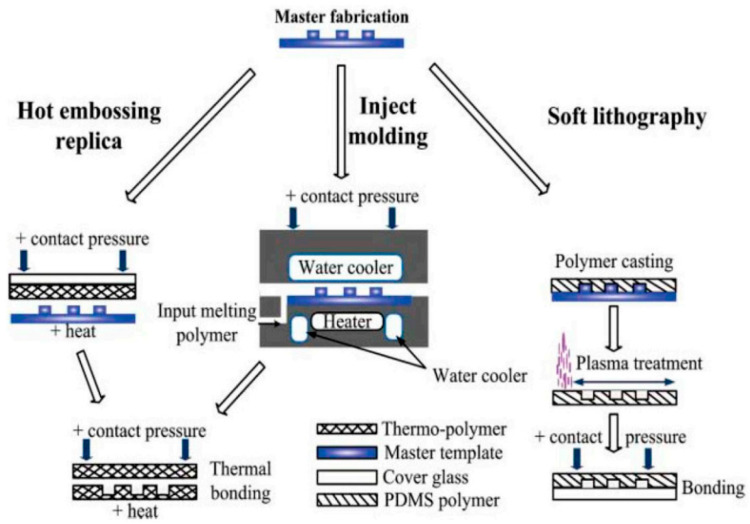
Representation of the fundamental molding methods for microfluidics fabrication. Reprinted from an open-access source [54]. PDMS: polydimethylsiloxane.

Soft lithography is one of the most popular methods for fabricating biomedical microfluidic devices [66]. Additionally, found in the literature under the name of “replica molding” [54], it allows the processing of elastomeric polymers and patterning of surfaces using PDMS stamps [20,70]. Soft lithography implies several steps—namely, creating the original hard master, pouring liquid polymer into the mold, heat-curing, and peeling off the polymer. In this way, a cast-molded stamp (replica) from a flexible material is created and further used for printing, molding, and embossing micro- and nanostructures [66,71,72]. A schematic of the soft lithography fabrication method, compared to similar molding techniques, is presented in Figure 2. The main advantages of this method are the obtaining of high-resolution replicas, the lower costs and more rapid production than the old photolithography method, the possibility to generate intricate 3D flows and pneumatic control lines by stacking multiple layers atop each other, and the ability to produce designs of high flexibility and high optical transmittance [54,72,73]. Concerning the limitations of the method, they are mostly related to the replica mold. Specifically, as the materials involved are soft, pattern deformation may occur, especially when removing the cast from the mold [54]. Another issue is that soft lithography is a semi-cleanroom process, implying costly photolithography techniques to realize the hard master [73].

#### 2.2.3. Laser-Based Processes

Generally, lasers are expensive tools, but compared to cleanroom facility costs, they are considered a more accessible fabrication technique [66]. Moreover, laser ablation supports the generation of microfluidic patterns on various materials in a rapid and flexible manner, without the hazards associated with chemical fabrication methods [63,64]. As a working principle, lasers optically amplify light via the stimulated emission of electromagnetic radiation [10]. A microstructure is created due to the thermal degradation effect, engraving the working material surface [66]. In more detail, short-duration laser pulses of controlled wavelengths break the chemical bonds from polymer molecules, while the associated rapid increase in temperature and pressure result in the ejection of decomposed polymer fragments. Hence, a photo-ablated cavity is produced [65,70,71]. The drawbacks of this method are related to the weak reproducibility caused by poor control of the laser focusing, undesirable surface effects, limited throughput, and product quality variations between different types of lasers [20,71].

Stereolithography is a classic 3D polymer structure-producing technique that fits under the umbrella of laser-based fabrication processes. This method is ideal for generating very fine features in a short time [54]. Liquid photopolymer resins are printed layer-on-layer and crosslinked with a focused laser or LED light source [74]. UV light is commonly used, but longer wavelengths can also be employed, depending on the photo-initiator type [54]. 

Two-photon polymerization is a technique through which structures are formed by curing arbitrary spots within the material [54]. Two-photon polymerization uses ultrashort laser pulses focused on a liquid resin volume to produce photopolymerization by two-photon absorption. The main disadvantage of two-photon polymerization is that voxels are cured one by one, consuming much time if the exposure area or the number of layers increases [19].

#### 2.2.4. Three-Dimensional printing

Three-dimensional printing is a relatively new, yet successful approach to forming microfluidic channels [17]. It ensures a precise application of materials to create a great variety of chip designs [10,35], especially for applications requiring complicated microfluidic structures [7]. The advantages that 3D printing brings have been exploited through several manufacturing technologies, such as fused deposition modeling, inkjet printing, multi-jet printing, and suspended liquid subtractive lithography [17,35]. However, some aspects limit the broad implementation of 3D printing in microfluidics manufacturing. The most important are the low z-resolution of printing systems, the limited variety of transparent materials, the need for extremely smooth surface finishes, and limitations in the precision fabrication of the hollow and void sections [75].

Fused deposition modeling, also found in the literature as freeform filament fabrication or extrusion-based additive manufacturing, is one of the most widespread 3D printing technologies [54,76]. Fused deposition modeling is based on the melting of a thermoplastic filament, followed by its extrusion through a nozzle and solidification by cooling [35]. The method is simple, effective, and affordable, allowing for multi-material printing [35,68]. However, some challenges must also be considered when deciding whether fused deposition modeling is suitable for specific microfluidic chips. The printed structures are more susceptible to compressive stress fractures due to inadequate fusion between adjacent layers [54]. Besides, the extruding filament sizes can be larger than the size of the microfluidic channels [76].

Multi-jet modeling, commercially known as PolyJet, is a promising 3D printing technology. Instead of a filament, a photosensitive resin is ejected as a droplet from the printing head and subsequently photo-cured by a light source attached to the inkjet printhead. Multi-jet modeling ensures high accuracy, being also able to build multi-material microfluidic platforms [54].

Inkjet printing was originally used to deposit colored inks, but it recently attracted interest for depositing materials such as metals, ceramics, polymers, and even tissues [59]. In this respect, inkjet printing is seen as a low-cost rapid alternative for chip production [73]. Other advantages of this fabrication method are simplicity, high precision, and high spatial resolution [59].

#### 2.2.5. Hybrid Technologies

Hybrid technologies appeared as a solution to overcome the challenges and limitations of each stand-alone fabrication method. For instance, Alapan et al. [75] combined 3D printing with micromachined laser lamination in order to obtain intricate transparent microfluidic devices. In this way, they eliminated the need for expensive and time-consuming cleanrooms while improving the design precision of the lamination process.

Another hybrid technology was proposed by Kojic et al. [68], who integrated the benefits of xurography and thermal lamination into a 3D prototyping printing process. Their fabrication method enabled rapid and robust manufacturing, with the potential of scaling-up the process by parallelizing the whole procedure.

Photolithography and thermal curing were brought together by Chen et al. [77] to create a low-cost, pump-free, capillary flow-driven microfluidic chip. The researchers obtained a two-positron emission tomography (PET)-layered device, with one of the layers containing microchannels formed by a UV-curable TE.

## 3. Applications of Microfluidic Devices

Microfluidic devices can be used in a plethora of applications, seeking to overcome the difficulties or challenges in traditional assays. The evidence shows great potential in personalized medicine, disease diagnosis, chemical screening, cell culture, cell separation, cell treatment, drug screening, drug delivery, and DNA sequencing [13,34,78,79]. Moreover, the particles that can be obtained in microreactors can be further used in diverse areas, ranging from electronics, energy, and textiles to biotechnology, bioimaging, biosensing, and gene delivery [80].

### 3.1. Diagnosis Devices

**Figure 3:**
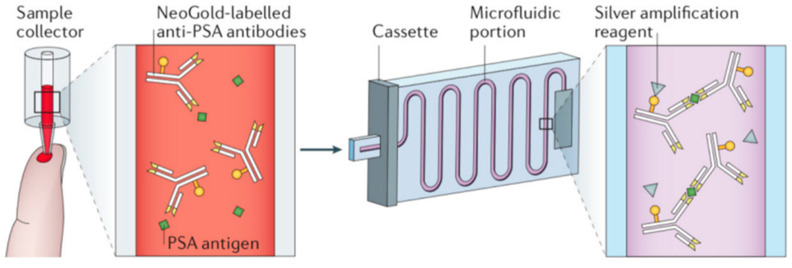
Example of a microfluidic diagnosis device that can detect prostate-specific antigen (PSA) in less than 15 min. Reprinted from an open-access source [82].

Microfluidic devices allow the analysis of various samples, such as blood, saliva, or cell tissues, to provide a rapid and accurate diagnosis [81] (Figure 3).

Microfluidic platforms have been constructed for microbial extraction and have been combined with several analytical methods to detect pathogenic microorganisms [83]. For instance, a microfluidic chip can capture airborne pathogens. By converting the laminar flow to a twisted airflow inside the device, the contact probability between the channel wall and the bacteria in the airflow is increased. Hence, the microfluidic platform can collect hundreds of bacteria within a couple of microliters of aqueous media, which is sufficient for direct immune analysis or nucleic acid analysis. However, this technique cannot work as itself, but it facilitates sampling and downstream bioanalysis [84].

Another substance that can be detected within microfluidic devices is creatinine. Its concentration is important in the determination of conditions like kidney failure, muscular dystrophy, and diabetic nephropathy. An example of this application was proposed by Narimani et al. [85], who created a cheap, portable, and efficient method to determine creatinine levels based on synthesized nanoparticles and colorimetric image-processing techniques. The device requires polyvinylpyrrolidone-coated silver nanoparticles and polyvinyl alcohol-coated silver nanoparticles, as different concentrations of creatinine will create color differentiations when mixed with the synthesized solution. The color changes were measured by capturing images in a designed isolated box with a uniformly illuminated imaging environment. MATLAB software applications allow real-time image processing, and the results are comparable with those from spectroscopic-based methods.

Point-of-care technologies can be used for the detection of hormones as well [86]. The most common example is represented by the paper-based microfluidics within home pregnancy tests. Their working principle is the detection of human chorionic gonadotropin hormone, a glycoprotein that starts increasing its concentration in urine after a missed menstrual period [87,88]. Nowadays, these tests are advanced enough that they can not only sense the presence of hormones in urine, but they can also quantify it. Depending on the hormone concentration, the duration of the pregnancy can be estimated and digitally displayed as “1–2 weeks”, “2–3 weeks”, and “3+ weeks” [89].

To monitor hydration and manage health disorders, Choi et al. [90] created a microfluidic platform that can capture, store, and analyze sweat biomarkers, rate, loss, and temperature. Their soft, skin-compatible, multimodal microfluidic device presented integrated color reference markers that provided accurate colorimetric estimates of analyte concentrations under different lighting conditions and in remote settings.

Microfluidic devices might also hold the answer for improving Coronavirus Disease 19 (COVID-19) testing. Amit Jadhav et al. [91] proposed a diagnosis protocol based on surface-enhanced Raman spectroscopy coupled with microchips presenting integrated microchannels functionalized with vertically aligned Au/Ag-coated nanotubes or with disposable electrospun micro/nano-filter membranes. Such constructions can successfully trap viruses from various biological fluids. Therefore, viruses may be accurately identified from their Raman signatures. However, this device is still in the prototype phase, and further investigations are required.

Just like the identification of pathogens or disease markers, DNA analyses can be performed on-chip to diagnose genetic-based diseases [34]. This can be achieved through Polymerase Chain Reaction (PCR)-integrated microchips. By controlling the reaction conditions and by introducing primers and other PCR reagents into the microfluidic platform, it can function as a point-of-care device for rapid and accurate analyses [83]. In this context, the use of microfluidic digital PCR has been employed for the detection of fetal chromosomal aneuploidy. Fan et al. [92] utilized uncultured amniocytes and chronic villus tissue, comparing the number of single-molecule amplification with a reference. The different target and reference chromosome counts allowed the identification of cases of fetal trisomy 21 (Down syndrome), trisomy 18 (Edward’s syndrome), and trisomy 13 (Patau syndrome) in less than six hours.

Le Roux et al. [93] developed a fully integrated chip for human identification by short tandem repeat analysis. The analysis comprises a unique enzymatic liquid preparation of the DNA, microliter noncontact PCR, and a high-resolution separation. The proposed microfluidic chip is completely self-contained, meaning that, after sample input, no other liquids can enter or exit the microchip during the assay. Moreover, the instrument itself is not directly in contact with any liquid. Therefore, the risk of contamination is minimized. Microfluidic devices for human identification are of great use also in forensics. COC chips can be employed for DNA amplification and the testing of samples in a simple, quick, and relatively sensitive manner. The best results can be acquired with sample quantities of less than one milligram or with a pure substance, and the result can be observed with the unaided eye [37]. Moreover, the DNA extraction chip can be coupled with an expert profile-interpretation software that allows law enforcement agencies to check whether there is a match between a person under custody and the DNA profiles recovered from unsolved crime cases [94].

### 3.2. Cell Culture Media

Since cells cultured in Petri dishes and tissue culture flasks undergo completely different environmental cues in comparison with natural tissues within a complex 3D ECM, miniaturized culture systems became a promising alternative [95]. Through accurately controlled fluid flows, microfluidic platforms can ensure relevant biochemical and biophysical cues to cultured cells in a well-defined and reproducible manner [96]. Therefore, it is possible to study tissue growth, renewal, and disease without difficulties specific to in vivo studies [95].

There is an increasing interest in developing microfluidic organs or tissues-on-a-chip, mainly due to two reasons: humans cannot be experimented on directly, and animal models may not mimic human physiology. Moreover, such devices may reduce investments and shorten the time for drug discovery and drug testing. Space and effort needed for animal testing are eliminated by the usage of organs-on-a-chip that can run in parallel on a single platform [81]. By lining up several such chips, each with different types of cells, the whole-body response can be replicated [79]. Organ-level functions can already be reproduced in microfluidic devices made of clear polymers with hollow microchannels containing cells [79]. The lungs, liver, and kidneys, among other organs, have attracted researchers’ interest in developing biomimetic microfluidic technologies [97].

Lung-on-a-chip devices are highly useful for examining the toxicity of several nanoparticles and understanding the pulmonary diseases that can result from the blockage of small air paths [98]. Chips that replicate the critical functional alveolar-capillary liquid/air interface are fabricated by growing alveolar epithelial cells and microvascular endothelial cells on different sides of a perforated silicone membrane [97].

Heart-on-a-chip is also a viable modeling possibility, allowing the analysis of contractility and electrophysiological behaviors in vitro. Besides, cardiac tissue contractility quantification can be performed under conditions of health, disease, and even exposure to chemical agents [98].

Liver-on-a-chip platforms can mimic in vivo conditions by recapitulating the sinusoidal structure of this organ, maintaining high cell viability and cellular phenotypes, and emulating the functions of native tissues [16]. In this respect, several models have been developed and are available for studying liver disease progression, facilitating drug discovery, and enabling toxicity tests [99]. For instance, Lee et al. [100] designed a liver test microfluidic platform using cell printing. The researchers provided the device with vascular and biliary channels to enhance liver functionalities in the chip.

**Figure 4:**
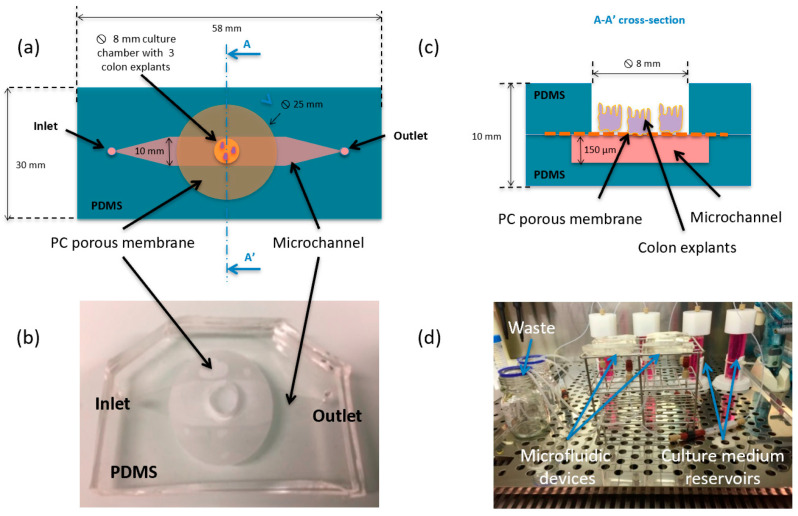
Gut-on-a-chip microfluidic device. (**a**) Schematic top view of the system made of two polydimethylsiloxane (PDMS) layers and one polycarbonate (PC) membrane; (**b**) current assembled device; (**c**) schematic drawing of the A-A’ cross-section; and (**d**) entire setup (including 4 devices, 9 colon explants, and 4 independent culture medium reservoirs and wastes) placed in an incubator. Reprinted from an open-access source [101].

Gut-on-a-chip microfluidic devices have also been developed [97], with one example offered by Baydoun et al. [101], who created a microfluidic mice colon culture model that can maintain the morphology of intestinal tissues for up to 192 h for a third of the explant (Figure 4).

**Figure 5:**
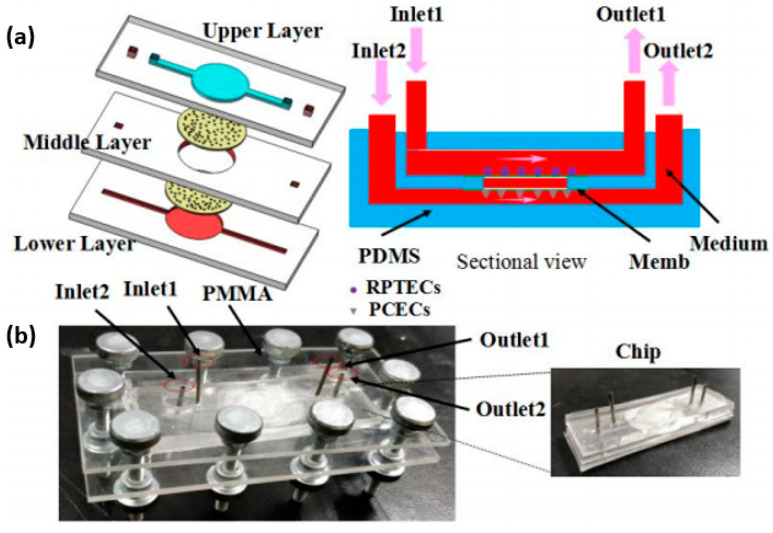
Kidney-on-a-chip microfluidic device. (**a**) Schematic diagram of the chip containing renal proximal tubular epithelial cells (RPTECs) and peritubular capillary endothelial cells (PCECs), and its sectional view and (**b**) current assembled chip. Reprinted from an open-access source [103].

Kidney-on-a-chip platforms have also attracted significant attention in the field of microfluidics, imitating the real renal tubular cell environment [102]. Yin et al. [103] built a three-layer microfluidic kidney chip that integrated PDMS microchannels and porous membranes, also developing a supporting microfluidic culture platform that enabled the long-term culture of renal cells (Figure 5). The researchers reported a better performance compared with cells cultured in Petri dishes, both in terms of cell growth and drug nephrotoxicity evaluation, rendering the device useful for preclinical studies.

Bone-marrow-on-a-chip is another microfluidic application that can lead to a better understanding of the lineage, commitment, and self-renewal of hematopoietic stem cells. Moreover, such models have the capacity to produce hematopoietic and immune cells in vitro, functioning as biosynthesis devices for generating various therapeutic agents. Furthermore, the bone marrow relation to radiation therapy allows the application of these tools in simulating and alleviating radiation-induced toxicity as well [15].

Even brain-on-a-chip models can be created to improve the in vitro drug evaluation process. These instruments hold the potential for creating a uniform profile of the controlled flow of nutrients, establishing individual cellular activity and providing a platform for the monitoring and excitation of neuronal cells. Besides, mechanical, physiological, pharmacological, and biochemical aspects can be studied in real-time through this technology [36]. Blood-brain barrier (BBB) replication on a microfluidic chip is also possible and highly useful. BBB-on-a-chip mimics the structure and complexity of the native BBB, allowing the investigation of central nervous system diseases [98].

Other opportunities for organs-on-a-chip involve the potential combinations between them to study complex mechanisms in disease and drug screening [38]. Tian et al. [104] developed a tissue-based liver–kidney-on-a-chip in which cell viability, tissue architecture, proliferation, and chemokine secretion were well-preserved. The authors used this multiorgan-on-a-chip to model the organotropism of breast cancer extracellular vesicles, obtaining similar results compared with animal models. However, the supreme goal is to create “human-on-a-chip”, a tool functioning as the whole body. Such a device has been called “homo chippiens” and opens the door for unprecedented pharmacokinetic and pharmacodynamic studies of experimental drugs [36].

Since cancer cells differ considerably in their response to therapy, drug tolerance, survival rate, and metastatic potential, this disease can be studied separately on so-called “cancer-on-a-chip” devices. These platforms are especially useful for drug testing and minimizing screening costs [81,98]. Moreover, by integrating these replicas of the tumor environment with different physiological modules, including the vasculature, cancer-on-a-chip models can further explore the interactions between cancer and other organs [15].

### 3.3. Drug Delivery Systems

The enormous potential of microfluidic devices has also become a great interest in the development of drug delivery systems [28,105]. The advantages of such microfluidic delivery systems are the precise dosage, targeted delivery, sustained and controlled drug release, the possibility of multiple dosing, and the appearance of only slight side effects [79]. Various cargos can be delivered via microfluidic systems, such as therapeutics, imaging modalities, and/or targeting moieties. With a theoretical encapsulation efficiency of 100%, microfluidics represents a fundamental shift towards advanced delivery system production [28].

**Figure 6:**
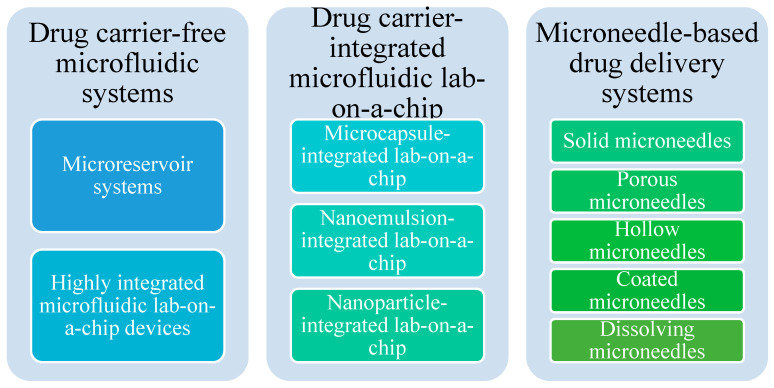
Microfluidic drug delivery systems classifications. Created based on information from a literature reference [105].

There are three main types of microfluidic delivery systems—namely, drug carrier-free microfluidic systems, drug carrier-integrated microfluidic lab-on-a-chip systems, and microneedle-based drug delivery systems [105]. Each class of devices may have subcategories, as presented in Figure 6.

One example of a carrier-free microfluidic system was offered by Kim et al. [106]. The researchers designed, fabricated, and tested a microfluidic intracochlear delivery system with a reservoir and active dose control. This approach resulted in a zero-net volume of liquid transfer while enabling the mass transport of compounds to the cochlea through diffusion and mixing. The system included a planar micropump (to generate reciprocating flow) and a drug reservoir (a long microchannel connected in a series with a micropump and parallel with the reciprocating flow network). The integrated device was tested on guinea pigs, leading to good results in terms of the safety and efficacy of the delivery. The authors were confident of the future use of their implantable cochlear drug delivery system for human clinical applications.

A carrier-integrated microfluidic chip was proposed by Gianella et al. [107], who created a multifunctional nanoemulsion platform for imaging-guided therapy. Their device could carry hydrophobic materials and could be used as a theranostic tool for simultaneous imaging-guided drug delivery in cancers. This two-fold goal could be achieved due to the oil-in-water nanoemulsions that could carry iron oxide nanocrystals for magnetic resonance imaging, fluorescent dye Cy7 for near-infrared fluorescence imaging, and hydrophobic glucocorticoid prednisolone acetate valerate for therapeutic purposes.

Microneedle systems are another good example of drug delivery via microfluidics [79]. Microneedles are minimally invasive devices that can access the microcirculation of the skin and deliver drugs through transdermal routes [105]. One such example was represented by biodegradable composite microneedles based on calcium sulfate and gelatin for the transdermal delivery of insulin created by Yu et al. [108]. The researchers reported less insertion pain and faster onset and offset of insulin pharmacokinetics in the body than for traditional subcutaneous administration. Moreover, the insulin released from the biodegradable microneedles had an effective hypoglycemic effect for a longer time compared with the subcutaneous injection route, holding great potential for diabetes treatment.

### 3.4. Nanomaterial Synthesis Platforms

It is undeniable that the development of nanotechnology has revolutionized many aspects of modern medicine, especially in the fields of biosensors, diagnostics, targeted drug delivery, and therapeutics [109,110,111]. Numerous nanotechnology-based pharmaceutical products have already been approved for clinical use, while many others are at different stages of preclinical development [112,113]. Microfluidic devices are excellent synthesis platforms for a wide range of nanoparticles, which, due to their narrow size distribution, uniform shape, improved reproducibility, and high encapsulation efficiency, can further serve in a plethora of applications [8,79].

Due to their unique physicochemical properties, such as a higher contrast or higher brightness than conventional small-molecule agents, nanoparticles synthesized in microfluidic devices are promising materials for fluorescence, magnetic resonance, and ultrasound imaging [8,97].

An example of materials appealing for nanomedicine is represented by iron oxide nanoparticles, which can be used as diagnostic and therapeutic agents against human disease. These non-toxic and biodegradable particles can act as contrast agents in magnetic resonance imaging or fluorescence imaging, drug carriers for small-molecule delivery, transfection vectors for gene therapy, and enhancers for magnetic hyperthermia [27]. Moreover, superparamagnetic iron particles can be employed in the on-demand release of active ingredients by magnetic positioning and exposure to an external stimulus, e.g., radiofrequency, heat, or in combination with responsive polymers for magnetic drug targeting [114].

Microfluidic reactors can also be employed to produce radioisotopes required in positron emission tomography (PET), a noninvasive medical diagnostic based on the intravenous injection of a drug with a known biological activity labeled with a positron-emitting nuclide. The most widely used drug of this sort is 2-[^18^F]-fluoro-2-deoxy-D-glucose (FDG), which is normally produced in batch processes in quantities sufficient for multiple doses from a single production run. However, FDG production is quite a complicated task, combining automation with computer science and involving specialized equipment, high costs, timescale planning, and personnel exposure. These challenges can be overcome by synthesizing FDG in microfluidic devices. Besides the general advantages of microfluidic synthesis over batch processes, in this case, it should also be mentioned that this technology is compatible with the dose-on-demand approach, allowing the production of a single dose of a tracer when a single PET scan is needed, with the possibility to restart synthesis whenever a new dose is required as well. As the reaction, purification, formulation, and quality control are all performed on a single small, disposable chip, the environmental impact is also reduced [115,116,117].

The delicate control of nano-synthesis achievable with microfluidic methods also allows for obtaining nanoparticles with higher sensing capacities and broader detection ranges than bulk materials. These properties serve well for creating biosensors [8]. One such material is polydiacetylene (PDA), which has a unique, naked eye-observable color switch and fluorescence enhancement in response to various external stimuli, e.g., solvent, pH, temperature, specific molecular recognition, and external forces. The interesting structural, spectral, and optical properties of PDA are considered only for self-assembled particles with controllable and uniform sizes. PDA sensors can be incorporated into low-cost, user-friendly devices, such as the smartphone-based differentiation of organic solvents, a wearable wristband for detecting dimethylformamide vapors, 3D-printed PDA/hydrogels for detoxification, and point-of-care devices for the diagnosis of ovarian cancer [118].

Other microfluidic synthesis products that can be used as biosensors are Au nanoparticles. Zheng et al. [119] developed a novel Au nanoparticles-based biosensor that can indicate different *Escherichia coli* concentrations and monitor the nanoparticle color changes via a smartphone application. In the first mixing channel of the chip, nanoparticles modified with capture antibodies, polystyrene microspheres modified with detection antibodies, and catalases are employed in the reaction with the target bacteria. Then, hydrogen peroxide is injected and catalyzed on the nanoparticle–bacteria–polystyrene complexes, which are captured in a separation chamber. After this, the mixture of Au nanoparticles and crosslinking agents is injected to react with the catalysate in the second mixing channel and is incubated in the detection chamber. There, Au nanoparticles aggregate, leading to a color changes from blue to red, which are is detected using the Hue–Saturation–Lightness-based imaging app on Android smartphones. Depending on this color, the concentration of the target bacteria is determined.

Microfluidics synthesis is also appealing for the pharmaceutical industry, as it allows the production of cheaper, more effective, and more accessible drug formulations [120,121]. Active pharmaceutical ingredients (APIs) that result from highly exothermic reactions can be obtained in microreactors, as is the case of nitroglycerin, an active agent used for acute cardiac infarction [120]. The enhanced control over the reaction, coupled with the quality of the products, convinced several pharmaceutical companies to implement microfluidic technology, especially for hazardous exothermic power-intensive syntheses [122,123]. Moreover, microfluidic devices are a valuable instrument for the encapsulation of water-soluble drugs in lipid nanoparticles in an effort to create more efficient drug formulations. In this respect, Hibino et al. [121] attempted to encapsulate coenzyme Q_10_ (CoQ_10_) in a MITO-Porter, a liposome for mitochondrial delivery. The researchers obtained homogeneously distributed, small-sized CoQ_10_-MITO-Porters that were efficiently internalized into cells and accumulated in the mitochondria. Other APIs reportedly produced within microfluidics are ibuprofen [123], lactose [124], aspirin [124], indomethacin [125], danazol [126], cefuroxime axetil [126], piroxicam [127], piracetam [127], carbamazepine [127], and more.

In addition, microreactors have been successfully employed in the fabrication of biodegradable polymer-based nanocarriers. One such example is poly(lactic-co-glycolic acid) copolymer (PLGA), a macromolecular compound approved by the Food and Drug Administration (FDA) that can be used for the synthesis of drug-loaded nanoparticles via a flow-focusing method in microchannels [128]. Chiesa et al. also managed to incorporate N-Acetylcysteine (N-Ac) in PLGA [129] and PLGA-PEG nanoparticles [130], obtaining promising drug carriers suitable for parenteral administration. The biocompatibility and biodegradability of these chosen polymers, together with precise control over the nanoparticle properties achieved through microfluidic fabrication, improved the in vivo biodistribution performances and N-Ac pharmacokinetic profile after administration. Other biodegradable polymers that have attracted research interest towards microfluidic production are poloxamer, chitosan, hyaluronic acid, alginate, and poly-2-vinylpyridine-b-poly(ethylene oxide) [131,132,133].

Much interest has also been drawn to the microfluidic platform synthesis of liposomes, as they represent highly efficient drug delivery systems [128]. Liposomal carriers achieve selective and sufficiently precise localization of the diseased site, also ensuring a slow and sustained release [134,135], the features required for the treatment of cancers and inflammatory conditions [136], infections, meningitis, malaria, HIV, hepatitis A, and influenza [137]. Liposomes of well-controlled sizes can encapsulate small molecules, such as amphotericin B and doxorubicin [138], being proposed to deliver vaccines, anticancer drugs, and gene therapy [134]. Solid lipid nanoparticles (SLNs) have also been widely evaluated as alternative drug delivery systems due to the possibility of a prolonged drug release and enhanced stability of the nanoparticle system. Producing SNLs by microfluidic methods results in superior properties in terms of size, polydispersity, and morphology compared to SNLs synthesized in bulk [139]. Niosomes represent a less-researched, yet equally promising, drug delivery platform. These nonionic surfactant-based vesicles can be prepared in microfluidic chips and be further used in food, cosmetic, and pharmaceutical applications [140]. In this respect, Ag Seleci et al. prepared a targeted niosomal drug delivery system to improve topotecan’s (TPT) therapeutic efficacy for gliomas by parallelly loading TPT synthesizing the controlled-sized niosomes [141].

Another sector in great need of microfluidic-produced nanoparticles is theranostics, an emerging field concerning the combination of diagnosis and treatment abilities into a single agent. Theranostics is especially useful for tackling cancer challenges. In this field, microfluidic systems contributed through their capability of multistep flow control to forming multifunctional nanoparticles bearing therapeutic and diagnostic agents with higher drug encapsulations in comparison to the classic methods [8,28,142]. An example of such particles is cancer cell membrane-coated nanoparticles, which consist of a nanoparticle core covered by a cancer cell plasma membrane coating that can carry tumor-specific receptors and antigens for cancer targeting. The core of these biomimetic nanoparticles can serve as a carrier for imaging and therapeutic moieties. The major applications for which these particles are used in cancer are homologous targeting to deliver imaging and therapeutic agents, the disruption of cancer cell–stromal cell interactions, and the induction of an immune response. However, some challenges and issues need to be solved before translating this therapeutic approach for use in humans [143]. Furthermore, quantum dots obtained through microfluidic methods are capable of diagnosing and delivering molecules to cancer cells in vivo. Through the control and maneuvering of reaction conditions in microfluidic synthesis, quantum dot functionality is increased, improving their sensitivity and ensuring the early detection of solid nodules or circulating tumor cell markers. As quantum dots hold great theranostic potential, academic research is moving towards clinical translation [144].

### 3.5. Emerging and Future Applications

Even though there are already many available applications, the development of microfluidics has just begun. The existing technology can be further improved, devices can be even more miniaturized, chips can be integrated with various other devices, synthesis processes can be better controlled, new reaction pathways can be investigated, and novel applications may arise.

The emerging technology is the creation of “template stickers” that can be selected and combined depending on the requirements of each reaction. A kit of standardized stickers is packed in a toolbox, each sticker representing one component of the final microfluidic chip. The fabrication process is mobile, inexpensive, and time-saving, while the resultant microfluidic devices have well-defined features, ideal performances, and customizability [7].

A distinct field that has begun to develop is flexible microfluidics, a multifunctional and multidisciplinary field that ingeniously combines biology, electronics, chemistry, and medicine [145]. The creation of flexible chips with reduced thicknesses and enhanced wear comfort opens the door for future use in smart contact lens sensors, the real-time noninvasive monitoring of physiological parameters, or tattoo-based sensors. Such applications are attractive for assessing the health status of astronauts, being capable of estimating their blood sugar, kidney function, and liver activity without impacting the overall mass of the spaceship [146].

Another area of opportunity for microfluidics is in pediatric patients or in patients suffering from conditions that make the sampling process difficult. For instance, microfluidic devices may be used to perform the sweat chloride test needed in cystic fibrosis. This is advantageous because it only requires a small-volume sample, which is a really important aspect, as the possible sample volume is limited by the low sweat volume and evaporation [147].

Another new and promising field has appeared at the convergence of microfluidics and ultrasounds. This combination is technically feasible, leading to synergistic results for drug encapsulation [3], diagnostics, and therapeutic applications [148].

Moreover, microfluidics can be involved in studying blood cell deformability to provide vital information for the early diagnosis of blood-related diseases. Besides, blood analog fluids can be analyzed in microfluidic devices, with the aim of developing new treatments in a personalized medicine approach [149].

Cells can not only be cultured and analyzed in microfluidic devices, but they can be created there as well. “Alive” artificial cells can be obtained with high throughput, easy operation, and precise control. As these biomimetic materials can imitate cell behaviors and act as bioreactors for synthetic biology, this emerging technology has great potential for cell function research, biomaterial fabrication, and regenerative tissue engineering [150].

Another synergistic convergence is the combined field of microfluidics and machine learning. Nowadays, most microfluidic devices are operated manually, but it is possible to develop and integrate on-chip multimodal instrumentation. In this way, autonomous platforms can be created, and the experimental data can be sent to machine learning for processing. Hence, instead of analyzing the results of the experiment after it is performed, machine learning allows the device to learn from the data and make accurate predictions to guide and optimize the conducted research. This intelligent microfluidics represents the next generation of platforms for drug discovery, nanomaterials, in vitro organ modeling, and developmental biology [151].

Several other innovative combinations of microfluidics with new domains, such as artificial intelligence, metamaterials, and neuromorphic engineering, may bring about unprecedented technological advancements in the foreseeable future [152].

## 4. Conclusions

To summarize, microfluidics technology represents an emerging multidisciplinary research field with extensive applications in various domains. The inexpensive, portable, and disposable nature of these chips makes them suitable for applications such as point-of-care devices, wearable biosensors, forensic tests, drug delivery systems, drug screening platforms, and microreactors for in situ preparations of various compounds.

The wide range of materials that are accessible nowadays, combined with the numerous possibilities for processing them, result in countless alternatives for fabricating microfluidic chips. By correlating and tailoring these two elements, it is possible to meet most (if not all) of the requirements coming from the market. Moreover, through a series of advancements in interconnected science fields, microfluidic devices have the potential to reach industrial-scale production.

To conclude, despite still being in its infancy, microfluidics has gained a lot of attention from researchers worldwide. Therefore, this field is expected to soon expand the knowledge of nanoparticle synthesis and nano- and biomedicine, pointing to disruptive applications that will solve some of the most pressing current healthcare problems.

## Figures and Tables

**Table 1:** Comparison of several available materials for the fabrication of microfluidic platforms. Adapted from the literature references [2,25,26,27,28,29,44,45,47,48].

Feature	Metal	Silicon	Glass	Ceramics	Elastomers	Thermoplastics	Resins	Hydrogels	Paper	Hybrids/Composites
Low cost	Positive	Negative	Negative	Positive	Moderate	Positive	Positive	Positive	Positive	Positive
Ease of fabrication	Positive	Negative	Negative	Positive	Positive	Moderate	Positive	Moderate	Positive	Moderate
Good mechanical properties	Positive	Positive	Positive	Negative	Positive	Positive	Positive	Moderate	Negative	Positive
Ease of sterilization		Positive	Positive	Negative	Positive	Positive	Positive	Negative	Negative	
Flexibility(Young’s modulus–GPa)	Negative (100–200)	Negative(130–180)	Negative(50–90)	Negative(65–250)	Positive (~0.0005)	Negative (1.4–4.1)	Negative (2.0–2.7)	Positive (low)	Positive (0.0003–0.0025)	
Oxygen permeability (Barrer)		Negative (<0.01)	Negative (<0.01)	Positive(>1)	Positive (~500)	Variable (0.05–5)	Negative (0.03–1)	Positive (>1)	Positive (>1)	Variable
Biocompatibility		Positive	Positive	Moderate	Positive	Positive	Positive	Positive	Positive	Positive
Chemical modification possibility		Moderate	Moderate	Moderate	Moderate	Moderate	Moderate	Positive	Moderate	Moderate
Optical clarity	Negative	Negative	Positive	Negative	Slight autofluorescence	Positive	Positive	Positive	Negative	Positive
Smallest channel dimension		<1 µm	<1 µm	>1 µm	<1 µm	<100 nm	<1 µm	>1 µm	>1 µm	
Low absorption		Positive	Positive	Positive	Positive	Positive	Positive	Moderate	Moderate	
Rapid prototyping		Moderate	Negative	Negative	Negative	Positive	Negative	Moderate	Moderate	Moderate
Tunable fluorescence	Negative	Negative	Negative	Negative	Positive	Negative	Negative	Moderate	Negative	
Potential for cell ingrowth	Negative	Negative	Negative	Negative	Negative	Negative	Negative	Positive	Positive	Negative

**Table 2:** The classification of microfluidic fabrication techniques.

	Material Removing Techniques	Material Depositing Techniques
Chemical processes	Electrochemical discharge machining [10] Wet etching [10] Dry etching [10]	Silicon surface micromachining [19] Lithography [60] Inkjet 3D printing [19] Powder 3D printing [19] Direct writing [19] Two-dimensional virtual hydrophilic channels [19]
Mechanical processes	Micro-milling [10] Micro-grinding [10] Micro-abrasive air-jet machining [10] Micro-abrasive water jet machining [10] Ultrasonic machining [10] Xurography [54]	Injection molding [54] Hot embossing [54]
Laser-based processes	Photothermal process [10] Ultra-short pulse process [10] Absorbent material process [10] Photochemical modification process [10] Laser direct machining [19]	Selective laser sintering [19] Stereolithography [54] Two-photon polymerization [54]
Other processes	Focused ion beam [10]	Forming process [10] Soft lithography [54,61] Layer-to-layer manufacturing [19] Layer-on-layer manufacturing [19] Fused deposition modeling [54] 2.5-Dimensional printing [19]

## Data Availability

Not applicable.
